# Electrospray-Based Microencapsulation of Epigallocatechin 3-Gallate for Local Delivery into the Intervertebral Disc

**DOI:** 10.3390/pharmaceutics11090435

**Published:** 2019-09-01

**Authors:** Moira Loepfe, Anja Duss, Katerina-Alexandra Zafeiropoulou, Oddny Björgvinsdóttir, Matteo D’Este, David Eglin, Giuseppino Fortunato, Juergen Klasen, Stephen J. Ferguson, Karin Wuertz-Kozak, Olga Krupkova

**Affiliations:** 1 Institute for Biomechanics, ETH Zurich, Hönggerbergring 64, 8093 Zurich, Switzerland; 2 AO Research Institute Davos, Clavadelerstrasse 8, 7270 Davos, Switzerland; 3 Empa, Swiss Federal Laboratories for Materials Science and Technology, Laboratory for Biomimetic Membranes and Textiles, Lerchenfeldstr. 5, 9014 St. Gallen, Switzerland; 4 Clinic Prodorso, Walchestrasse 15, 8006 Zurich, Switzerland; 5 Schön Clinic Munich Harlaching, Spine Center, Academic Teaching Hospital and Spine Research Institute of the Paracelsus Medical University Salzburg (AU), Harlachinger Str. 51, 81547 Munich, Germany; 6 Department of Health Sciences, University of Potsdam, Am Neuen Palais 10, 14469 Potsdam, Germany

**Keywords:** degenerative disc disease, inflammation, drug delivery, EGCG, microparticles, injectable biomaterial, electrospraying

## Abstract

Locally delivered anti-inflammatory compounds can restore the homeostasis of the degenerated intervertebral disc (IVD). With beneficial effects on IVD cells, epigallocatechin 3-gallate (EGCG) is a promising therapeutic candidate. However, EGCG is prone to rapid degradation and/or depletion. Therefore, the purpose of this study was to develop a method for controlled EGCG delivery in the degenerated IVD. Primary IVD cells were isolated from human donors undergoing IVD surgeries. EGCG was encapsulated into microparticles by electrospraying of glutaraldehyde-crosslinked gelatin. The resulting particles were characterized in terms of cytocompatibility and anti-inflammatory activity, and combined with a thermoresponsive carrier to produce an injectable EGCG delivery system. Subsequently, electrospraying was scaled up using the industrial NANOSPIDER™ technology. The produced EGCG microparticles reduced the expression of inflammatory (IL-6, IL-8, COX-2) and catabolic (MMP1, MMP3, MMP13) mediators in pro-inflammatory 3D cell cultures. Combining the EGCG microparticles with the carrier showed a trend towards modulating EGCG activity/release. Electrospray upscaling was achieved, leading to particles with homogenous spherical morphologies. In conclusion, electrospray-based encapsulation of EGCG resulted in cytocompatible microparticles that preserved the activity of EGCG and showed the potential to control EGCG release, thus favoring IVD health by downregulating local inflammation. Future studies will focus on further exploring the biological activity of the developed delivery system for potential clinical use.

## 1. Introduction

Degenerative disc disease (DDD), a pathology of the intervertebral disc (IVD), is associated with inflammation, premature senescence, and cell death, all of which contribute to the loss of extracellular matrix (ECM) and the development of discogenic back pain [1,2,3]. Our previous research showed that the polyphenol epigallocatechin 3-gallate (EGCG), found, e.g. in tea plants (*Camelia sinensis*), can inhibit these hallmarks, thus representing a promising therapeutic candidate to combat the loss of IVD function. EGCG interferes with the pro-inflammatory IL-1β cascade by reducing the activity of IRAK1–NF-κB/JNK/p38 signaling and subsequent expression of inflammatory and catabolic genes, namely interleukins (IL-6, IL-8), matrix metalloproteinases (MMP-1, MMP-3, MMP-13), toll-like receptor 2 (TLR-2), cyclooxygenase 2 (COX-2), nerve growth factor (NGF), and inducible nitric oxide synthase (iNOS) [4,5]. Moreover, the local application of EGCG attenuates radiculopathy in a rat model of disc herniation, hence supporting the clinical relevance of this compound [4]. In addition to its anti-inflammatory effects, EGCG also inhibits oxidative stress-induced death of IVD cells by activating the PI3K/Akt pathway and protecting mitochondrial membranes from depolarization [6]. As cytokines play an important role in IVD homeostasis [7], their natural (balanced) levels should be preserved. It is well documented that some commonly used therapeutics (e.g., corticosteroids) strongly interfere with cytokine synthesis and release [8], thereby significantly inhibiting cytokine-regulated pathways, including those involved in normal cell metabolism, proliferation or exo-/endocytosis [9,10]. Given its mechanism of action, EGCG might be able to preserve natural (low) levels of cytokines and ECM enzymes, thus reducing the potentially damaging side effects in the IVD.

Oral administration is not ideal for the application of EGCG in the treatment of DDD. It has been shown that only 0.1–1.1% of the orally administered EGCG reaches the systemic circulation [11]. On the other hand, high doses of EGCG-containing preparations (10–29 mg/kg/day) can cause hepatotoxicity and nephrotoxicity, both in animal models and in humans [12,13]. As the IVD has a limited blood supply, it remains unknown how much of an ingested EGCG can actually reach the inner disc tissue (nucleus pulposus = NP). Local drug delivery (e.g. intradiscal injection) can help to overcome these issues and significantly lower the risk of systemic side effects [14]. A delivery system with the ability to provide sustained release, prolong activity, and account for specific local tissue requirements might be necessary for the therapeutic application of active EGCG in the IVD.

Various encapsulation techniques for polyphenols have been developed [14]. However, these techniques might not be suitable for an EGCG molecule that degrades under the harsh conditions (pH, temperature) common to traditional encapsulation techniques [13,15]. Electrospraying is a gentle electrohydrodynamic encapsulation method that may overcome these limitations, as it can generate solid particles in a one-step process without the need for high temperatures or toxic solvents. Due to the latest improvements in electrospraying protocols, it is now possible to produce particles with controlled sizes and size distributions at much faster production rates [16]. Recently, electrosprayed EGCG-loaded gelatin microparticles with enhanced EGCG stability were prepared for food-grade applications [17]. Therefore, the overall goal of this study was to explore electrospraying-based controlled delivery of EGCG as a potential therapeutic method for the treatment of DDD. We hypothesized that the encapsulation of EGCG by electrospraying will result in cytocompatible microparticles with preserved EGCG activity. We further hypothesized that combining EGCG microparticles with a suitable carrier will facilitate minimal invasive intradiscal delivery and further modulate/prolong EGCG activity/release.

## 2. Materials and Methods

### 2.1. Materials

Gelatin (porcine skin gelatin type A, G1890 Sigma, St. Louis, MI, USA) at 4% *w/v* or 6% *w/v* was mixed with 20% *v/v* acetic acid (33209, Sigma) in H_2_O, stirred for 4–6 h at 40 °C and subsequently cooled down to room temperature [18,19]. Non-crosslinked gelatin, as well as gelatin crosslinked with glutaraldehyde (GA, G6257 Sigma), were used. A stock solution of glutaraldehyde was prepared as 5% *w*/*v* in PBS. Three different concentrations of GA (37.5 μg/mL, 62.5 μg/mL and 87.5 μg/mL) were prepared from stock GA solution by pipetting into gelatin solution and instant mixing [20]. A total concentration of 5 mM (2.293 mg/mL) EGCG (E4143, Sigma) was chosen, aiming for a long-term sustained release of 10–100 µM EGCG. 0.9% Sodium chloride solution (7647-14-5, Fisher chemical, Reinach, Switzerland) in H_2_O was used as a release medium for ferrous tartrate and DPPH assays. Hyaluronic acid-Poly(*N*-isopropylacrylamide) (HA-pNIPAM) was synthesized as previously reported [21]. To prepare 15% *w/v* HA-pNIPAM in H_2_O, HA-pNIPAM powder was weighed in two steps with a drying period at 37 °C in between (to reduce the absorption of water from the air during weighing and to maintain consistency in the hydrogel properties), and was slowly dissolved overnight at 4 °C.

### 2.2. Experimental Workflow 

**Figure 1:**
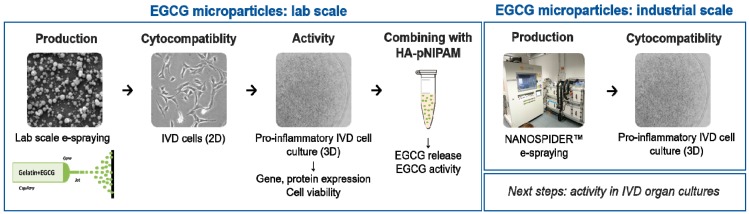
Experimental workflow. The polyphenol epigallocatechin 3-gallate (EGCG) microparticles were produced by electrospraying (e-spraying) and their cytocompatibility was tested in intervertebral disc (IVD) cells cultured in 2D. The activity of EGCG microparticles was tested in pro-inflammatory IVD cell cultures utilizing 3D alginate beads treated with 5 ng/mL IL-1β for seven days. Next, the particles were combined with a thermoreversible HA-pNIPAM carrier and the activity and release of EGCG were measured. Then, the production of EGCG microparticles was upscaled and cytocompatibility of the resulting particles was measured in the pro-inflammatory IVD cell cultures, utilizing 3D alginate beads treated with 5 ng/mL IL-1β for seven days.

The experimental workflow is outlined in the below schematic (Figure 1).

### 2.3. Electrospraying

Electrospraying was performed using a custom-made setup. The syringe was connected to a high voltage supply and situated within a climate chamber for environmental control, with the temperature and humidity being fixed to 24 °C and 40%, respectively [22]. Gelatin was introduced in a 5-mL plastic syringe and pumped at a steady flow rate and voltage through a 20 G stainless-steel needle. Microparticles were collected on a grounded aluminum sheet placed 10 cm from the spray needle [20,23]. The formation of spherical particles was achieved by varying the electrospraying parameters, namely flow rate (2 or 4 µL/min), voltage (20–22 kV), and concentration (4–6%) [24]. GA and EGCG were both added on the day of spraying. The collected microparticle powder was stored at –20 °C in glass vials, sealed, and protected from the light. Drug loading capacity (LC) was calculated as follows: EGCG LC [%] = weight of the drug in particles/weight of particles × 100.

### 2.4. Electrospraying Scale-Up

As laboratory-scale electrospraying generated relatively low amounts of particles, upscaling was necessary to approach realistic batch sizes. The particles were electrosprayed using the needle-free NANOSPIDER™ technology, one of the first industrial-scale devices for electrospinning (Elmarco, NS Production Line NS 1S500U, Liberec Czech Republic) [25,26]. During the NANOSPIDER electrospraying, parameters such as voltage, temperature, incoming light intensity, cylinder diameter, wire speed (electromagnetic wire speed = EMW, rotational wire speed = RW), electrode distance to the collector, and humidity were optimized. To improve the surface tension, 0.1% Tween20 (P1379, Sigma-Aldrich, St. Louis, MI, USA) in H_2_O, a biocompatible surfactant, was added [27]. GA and EGCG were both added on the day of spraying. The particles were collected immediately after the spraying and stored as described above.

### 2.5. Scanning Electron Microscopy

The particles were visualized by a scanning electron microscope (SEM) (Hitachi SU5000, Tokyo, Japan). Representative samples from the border as well as from the center of the sprayed area were taken from each batch. The samples were platinum/palladium (80:20) sputtered using CCU-010 Safematic (Bad Ragaz, Switzerland) in an argon atmosphere at room temperature to achieve a coating thickness of 10 nm. At least five pictures were taken from each sample, to check for local differences. 

Pictures were taken at 5×, 10×, and 100× magnification. Images were analyzed with ImageJ/Fiji, where the diameter of 10 particles per image was measured using the function “measure size” and averaged for each sample (>100 particles in total).

### 2.6. Ferrous Tartrate Assay (EGCG Release)

The release of EGCG was measured in 10 mL of 0.9% NaCl (release medium) over a period of seven days. The release medium was collected 1, 24, 72, 120, and 168 h after starting the release experiment and replaced by an equivalent amount of fresh release medium. The released EGCG was measured using the ferrous tartrate assay, as described before [28]. Briefly, 150 µL of sample were mixed with an equal amount of ferrous tartrate solution consisting of 1 g ferrous sulfate (FeSO_4_, Sigma, F8633) and 5 g potassium sodium tartrate tetrahydrate (KNaC_4_H_4_O_6_, Sigma, S2377) in 1000 mL distilled water mixed with 0.067 M potassium phosphate buffer (pH 7.5) at a ratio of 1:4. Colorimetric spectroscopy of 100 µL of each sample solution mix was performed at 540 nm in transparent 96-well BRANDplates (Huberlab, Aesch, Switzerland) using a plate reader (Tecan, Infinite M200 PRO, Männedorf, Switzerland). EGCG concentrations were calculated from a standard curve. The cumulative release was calculated from the obtained data. The Korsmeyer–Peppas model was used to determine the EGCG release kinetics under sink conditions, with an initial EGCG concentration higher than the matrix solubility, and assuming constant EGCG diffusivity and negligible swelling of the matrix. The sink conditions were achieved by ensuring the concentration of the released drug in the release medium never reached more than 10% of its saturation solubility [29,30].

### 2.7. DPPH Radical Scavenging Assay (EGCG Activity)

The antioxidant potential of released EGCG was evaluated by measuring its ability to scavenge the free 2,2-diphenyl-1-picrylhydrazyl (DPPH) radical as described before [6]. Briefly, 100 μL of the sample was incubated with 500 μL of DPPH (250 μM in ethanol, ChemCruz, sc-202591, Huissen, The Netherlands) for 1 h at room temperature in the dark. Absorbance was measured at 517 nm in transparent 96-well BRANDplates (Huberlab). l-Ascorbic acid (Sigma, A4544) and ethanol (Merck, 100983, Kenilworth, NJ, USA) were used as positive and negative controls, respectively. DPPH radical scavenging activity [%] was calculated as [(absorbance of the negative control − absorbance of the samples)/absorbance of the negative control × 100].

### 2.8. Drug Delivery System

Due to their potential mechanical/chemical instability, gelatin microparticles (*n* = 4 batches) were embedded in a 15% HA-pNIPAM hydrogel to create a thermoresponsive delivery system (=EGCG particles in the hydrogel group). HA-pNIPAM was synthesized as reported in [21]. The hydrogel was hydrated with 1 mL H_2_O and kept overnight at 4 °C to complete solubilization. On the next day, 42.3 mg particles containing EGCG (5 mM) were added to HA-pNIPAM (1 mL) at room temperature (liquid state) and then heated up to 37 °C (gelation). Free 5 mM EGCG encapsulated in HA-pNIPAM (=free EGCG in the hydrogel group), as well as particles without the hydrogel (=EGCG particles group), were tested as controls.

### 2.9. Donors and IVD Cell Culture

Human IVD tissue was obtained with informed consent from donors undergoing IVD surgeries (*n* = 10). The study was approved by the Kantonale Ethikkommission Zürich (01/2009, 05/2019 EK-16/05; 2019-00736, 01/2009, 05/2019). The tissues were enzymatically digested as described before [4], and liberated primary cells were then seeded in Dulbecco’s Modified Eagle’s Medium (DMEM/F12, D8437, Sigma) supplemented with 10% fetal calf serum (FCS, F7524, Sigma), and 1% Anti/Anti (15240-062, Gibco, Waltham, MA, USA). The cells were sub-cultured using 1.5% trypsin (15090-046, Gibco) in a standard cell culture incubator (37°C, 21% O_2_, 5% CO_2_) [4]. IVD cells in passage two or three were used for experiments.

### 2.10. Cytocompatibility Test

Due to the potential toxicity of the solvent (AA) and the crosslinker (GA), the cytocompatibility of plain gelatin microparticles crosslinked with GA (62.5 μg/mL) was tested by MTT assay as described before [6]. Briefly, 100,000 disc cells/well (*n* = 4 donors) were seeded in 12-well plates and incubated overnight. The next day, 7.5, 15, and 30 mg/mL of sterilized gelatine particles were placed in the upper part of a trans-well system (ThinCert™ Tissue Culture Inserts, 82051-570, VWR, Radnor, PA, USA) immersed in culture media. After four days, 0.5 mg/mL fresh 3-[4,5-dimethylthiazol-2-yl]-2,5-diphenyl tetrazolium bromide (MTT, M5655, Sigma) was added and maintained for 3 h at 37 °C. Finally, MTT was discarded, cells were lysed in DMSO (D8418, Sigma) and the absorbance was measured at 565 nm as described before [6]. The results are shown relative to control cells (= 100% viable).

### 2.11. Three-Dimensional (3D) Disc Disease Model

To create a three-dimensional (3D) disc disease model, IVD cells isolated from one donor were seeded in 1.2% alginate (71238-50G, Sigma) at 4 × 10^6^ cells/mL alginate as described previously [4]. Briefly, the cells–alginate mixture was dropped into 102 mM calcium chloride solution (1.02382, Merck) using a sterile syringe and 21G needle and left for 8 min to polymerize under gentle stirring. The resulting beads were washed with 0.9% NaCl (1.06404, Merck) and PBS, equally distributed into six-well plates and pre-cultured for seven days in DMEM/F-12 supplemented with 10% FCS, 3% Anti-Anti, and 50 µM ascorbic acid 2-phosphate (A8960, Sigma), to support the production of the native ECM. On day 7, the medium was exchanged (to complete DMEM/F-12) and the beads were stimulated with 5 ng/mL IL-1β. IL-1β significantly reduces the gene expression of matrix molecules (aggrecan, collagen) and upregulates catabolic enzymes (ADAMTS4, MMP1, MMP3, MMP13) and inflammation mediators (IL-6, IL-8, NGF, COX-2), thus providing conditions that mimic naturally occurring DDD [4,31]. In the first experiment, IL-1β was used alone or in the presence of EGCG microparticles (three repeats, 21 mg per 9 mL of DMEM/F-12) placed at the bottom of the cell culture plates. In the second experiment, IL-1β was used alone or in the presence of two EGCG formulations placed in the transwells: (1) free EGCG in the hydrogel and (2) EGCG microparticles in the hydrogel (=drug delivery system). The hydrogel-only group was tested as well. After seven days of pro-inflammatory/catabolic culture, the medium was collected, cell viability was determined, and the cells were harvested for RT-qPCR.

### 2.12. Cell Viability in 3D (Live/Dead)

The viability of IVD cells seeded in the 3D alginate beads was evaluated at the end of the experiment by Live/Dead staining as described previously [4]. Briefly, 2 µM ethidium homodimer (46043, Sigma) and 2 µM calcein-AM (17783, Sigma) in DMEM/F-12 were applied to one bead per well per condition at 37 °C in the dark. After 30 min, the beads were gently squeezed between cover slips and three photos were randomly captured with a fluorescence microscope (Olympus IX51, Tokyo, Japan) at the wavelength of 515 nm (calcein: living cells) and 620 nm (ethidium: dead cells). Image J was used to count the number of viable and dead cells.

### 2.13. Cytokine Release (ELISA)

The culture medium was collected and analyzed for IL-6 and IL-8 using enzyme-linked immunosorbent assay (ELISA) kits according to the manufacturer’s protocols (BD Biosciences 555220 and 555244, San Jose, CA, USA) with human recombinant IL-6 or IL-8 as the standard. IL-6 and IL-8 concentrations were calculated based on the standard curve and shown as pg/mL.

### 2.14. Gene Expression Analysis (RT-qPCR)

Cells were liberated from the beads by 55 mM Sodium citrate solution [55 mM Sodium citrate tribasic dihydrate (71406, Sigma) + 150 mM NaCl (1.06404.100 Merck) + 10 mM EDTA (E4884, Sigma) in H_2_O and lysed with 350 µL RLT buffer (in RNeasy kit). RNA was isolated using the RNeasy Mini kit (74106, Qiagen, Venlo, The Netherlands) according to the manufacturer’s recommendations. The purity and concentration of the resulting RNA were measured using the NanoDrop (ND-1000, Thermo Fisher Scientific, Waltham, MA, USA). One milligram of total RNA was reverse-transcribed to cDNA using a reverse transcription kit (4374966, Applied Biosystems, Foster City, CA, USA). qPCR targets were selected based on previous studies on EGCG in the disc [4,5]. qPCR was performed using a mixture of primers/probes (Table 1) and master mix (4367846, Applied Biosystems) on the CFX96 Real-Time System (Bio-Rad Laboratories, Hercules, CA, USA). The relative expression level was calculated by the ΔΔ*C*t method. For normalization purposes, samples with undetectable expression were assigned a *C*t value of 40 [32]. The results are shown as the fold change relative to control.

### 2.15. Statistical Analysis

Statistical analysis was performed in GraphPad, Prism 8.0.0 (San Diego, CA, USA). Multiple groups were compared using the Kruskal–Wallis test with Dunn’s post hoc test, after testing for data normality (Shapiro–Wilk test). Two groups were compared using the *t*-test. Results are shown as mean ± SD. *p* < 0.05 was considered statistically significant (* *p* < 0.05, ** *p* < 0.01, *** *p* < 0.001).

## 3. Results

### 3.1. Optimization of Electrospraying

**Figure 2:**
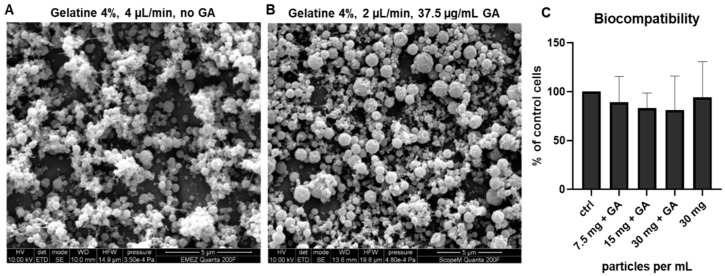
Optimized electrospraying of gelatin microparticles. (**A**) SEM images of plain gelatin particles sprayed with 4% *w*/*v* gelatin under 4 μL/min flow rate (*n* = 7 batches). (**B**) SEM images of glutaraldehyde (GA)-crosslinked particles sprayed with 4% *w/v* gelatin and 37.5 μg/mL GA under a 2 μL/min flow rate (*n* = 7 batches). (**C**) Biocompatibility of GA–gelatin microparticles after four days of indirect contact with disc cells (*n* = 4 donors, Kruskal–Wallis with Dunn’s post hoc test, *p* < 0.05).

The optimization of electrospraying was performed by adjusting the voltage (20–22 kV), gelatin concentration (4% or 6%), flow rate (2 and 4 µL/mL), and GA concentration (37.5, 62.5, 87.5 µg/mL). The 6% gelatin solution caused fiber formation, especially at higher flow rates. As expected, fiber formation was reduced in the 4% gelatin solution at lower flow rates (Figure S1). Based on the optimizations, protocols describing the small-scale production of the plain (non-crosslinked) and GA-crosslinked gelatin microparticles were established. Final plain gelatin particles (used further in the cytocompatibility test) were sprayed using non-crosslinked 4% *w*/*v* gelatin at a 4 μL/min flow rate and 21 kV voltage, with a resulting average particle diameter of 476 ± 125 nm (*n* = 7 batches) (Figure 2A). Final GA–gelatin particles were sprayed using 4% *w/v* gelatin crosslinked with 37.5 μg/mL GA under a 2 μL/min flow rate and 20 kV voltage, with a resulting average particle diameter of 669 ± 172 nm (*n* = 7 batches) (Figure 2B). Both types of particles had a uniform spherical shape and were equally distributed on the collector substrate without clumps or fibers. The difference in gelation/solubility between the cross-linked and non-crosslinked microparticles was monitored by immersing the particles in culture media for 24 h at 37 °C. Plain gelatin microparticles were dissolved after 2 h, while microparticles crosslinked with 37.5 μg/mL GA swelled at the 24 h time point, indicating enhanced stability (not shown). Both plain and GA-crosslinked gelatin microparticles were further used for cytocompatibility testing. The cytocompatibility was analyzed by MTT assay using IVD cells cultured for four days in the presence of plain and crosslinked (62.5 μg/mL GA) particles. Despite a trend towards reduced cell viability with the increasing concentration of GA, no significant changes were detected (Figure 2C). As a lower GA concentration (37.5 μg/mL) is used in the final protocol, the particles are expected to be nontoxic for disc cells.

### 3.2. Encapsulation of Epigallocatechin 3-Gallate (EGCG)

**Figure 3:**
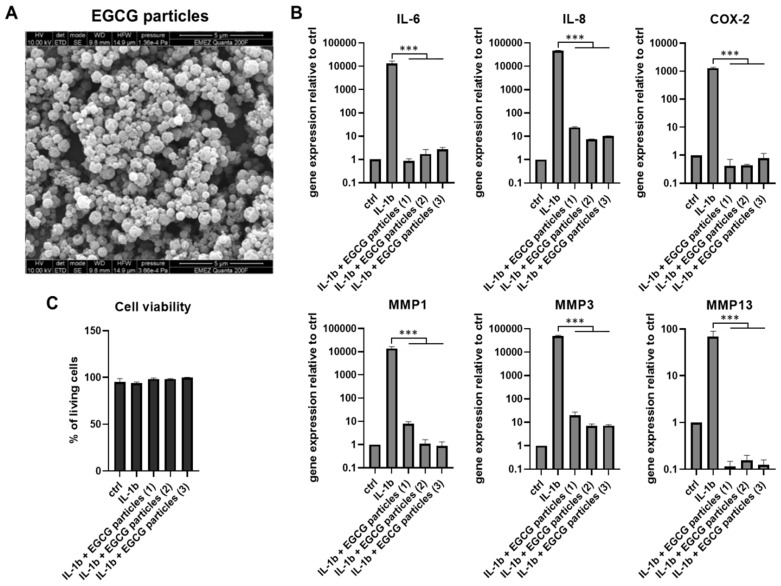
Epigallocatechin 3-gallate (EGCG) encapsulated in microparticles. (**A**) Representative SEM image of EGCG particles produced by electrospraying (*n* = 7). (**B**) Gene expression of inflammation and catabolic mediators (IL-6, IL-8, COX-2 MMP1, MMP3, MMP13) in disc cells cultured in 3D for seven days in the presence of 5 ng/mL IL-1β with and without EGCG microparticles (*n* = 3 repeats labelled as particles (1), (2) and (3), *t*-test IL-1β vs. IL-1β + EGCG particles, * *p* < 0.05, ** *p* < 0.01, *** *p* < 0.001). (**C**) Cell viability of EGCG-gelatin microparticles after seven days of indirect contact with disc cells cultured in 3D (*n* = 3 repeats, Kruskal–Wallis with Dunn’s post hoc test, * *p* < 0.05, ** *p* < 0.01, *** *p* < 0.001). ctrl = untreated cells.

The EGCG–gelatin particles were sprayed using 4% *w*/*v* gelatin crosslinked with 37.5 μg/mL GA under the established conditions: 2 μL/min flow rate, 20 kV voltage, 10 cm electrode distance, 40% relative humidity, 24 °C and in the dark (due to light sensitivity of EGCG). The resulting average particle diameter was 661 ± 120 nm (*n* = 7 batches) (Figure 3A). The presence of EGCG in the polymer solution during electrospraying did not have any effect on the particle formation process and/or size when compared with particles without EGCG. The drug loading capacity (LC) of produced microparticles was calculated at 5.42 wt % (for 5 mM EGCG and 4% *w*/*v* gelatin).

### 3.3. Biological Activity of EGCG Microparticles 

The effects of electrospraying on the biological activity of EGCG were assessed in the pro-inflammatory 3D cell culture model (alginate beads). The beads were divided into equal groups to be stimulated with 5 ng/mL IL-1β alone or in the presence of EGCG microparticles (in three repeats of one batch marked as EGCG particles 1, 2, 3). The IL-1β-only group was then compared with the IL-1β+EGCG particles groups. Non-stimulated alginate beads were used as the control (ctrl). After seven days in the presence of 5 ng/mL IL-1β, EGCG microparticles significantly reduced the gene expression of inflammation and catabolic mediators IL-6, IL-8, COX-2, MMP1, MMP3, and MMP13 (Figure 3B). The protein release of IL-6 and IL-8 into culture media was reduced as well, which confirmed the gene expression results (Table 2, Figure S2) and suggested that electrospraying encapsulation preserved the biological activity of EGCG. The cell viability of all tested groups was comparable to the control and close to 100%, indicating that the EGCG microparticles were cytocompatible in the presence of inflammation.

### 3.4. EGCG Drug Delivery System

**Figure 4:**
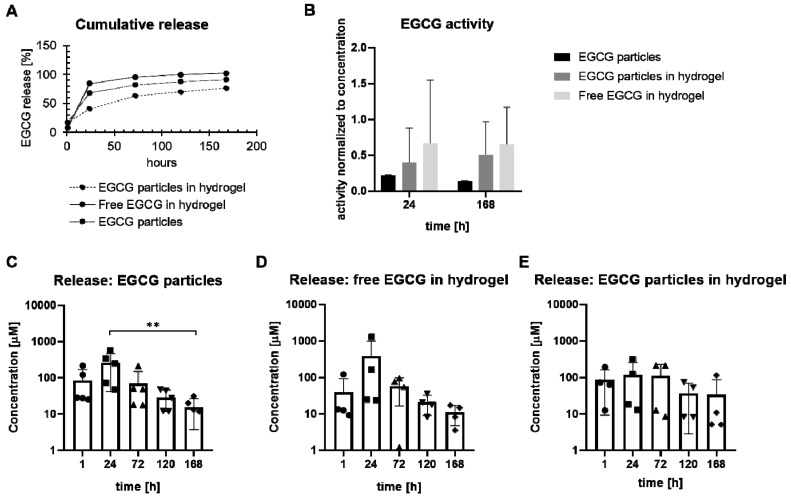
Epigallocatechin 3-gallate (EGCG) drug delivery system. (**A**) Cumulative release of EGCG from microparticles alone, EGCG particles in the hydrogel (HApNIPAM), and free EGCG in the hydrogel (*n* = 4). (**B**) Relative activity of released EGCG on day 1 and 7 (*n* = 3). (**C**–**E**) Seven-day release experiment with (**C**) EGCG particles only, (**D**) free EGCG in the hydrogel, and (**E**) EGCG particles in the hydrogel (*n* = 4). Kruskal–Wallis with Dunn’s post hoc test, * *p* < 0.05, ** *p* < 0.01, *** *p* < 0.001.

Unprotected microparticles might migrate and escape the IVD space and/or be mechanically damaged upon injection into the disc. Therefore, EGCG microparticles were additionally dispersed in the in situ-forming hydrogel HA-pNIPAM and the release of EGCG from the drug delivery system (*n* = 4 batches) was monitored for one week. The EGCG particles alone and fresh free EGCG in HA-pNIPAM were used as reference. At the 24 h time point, most EGCG was released in the free EGCG in the hydrogel group (~80% of the encapsulated dose) followed by the EGCG-particles-only group (~65%). The drug delivery system (EGCG particles in the hydrogel group) tended to slow down the release (~40% at the 24 h time point) (Figure 4A). EGCG incorporated in microparticles showed a high-dose release during the first 24 h (Figure 4C). Free EGCG in HA-pNIPAM showed a similar trend (Figure 4D), demonstrating that improvement in EGCG retention in HA-pNIPAM through encapsulation may be needed. The release of EGCG from microparticles in the drug delivery system tended to be more uniform at the 24 h time point (Figure 4E). According to the Korsmeyer–Peppas model, EGCG was transported through the gelatin microparticles via diffusion in the EGCG particles group (Fickian diffusion, *n* = 0.39). The free EGCG in the hydrogel group fitted best with a non-Fickian diffusion, *n* = 0.62 (Figure S3). The multi-component EGCG particles in the hydrogel group (consisting of two materials with possibly diverse physicochemical properties) exhibited a more complex release pattern (*n* = 0.27), possibly due to a combination of erosion and/or swelling and deviation from the model prerequisites. The activity of released EGCG was comparable in all three groups (Figure 3B). Interestingly, the EGCG activity tended to be higher in both HA-pNIPAM groups (free EGCG and EGCG particles), suggesting that HA-pNIPAM may possess drug-protective properties.

### 3.5. Electrospraying Scale-Up

**Figure 5:**
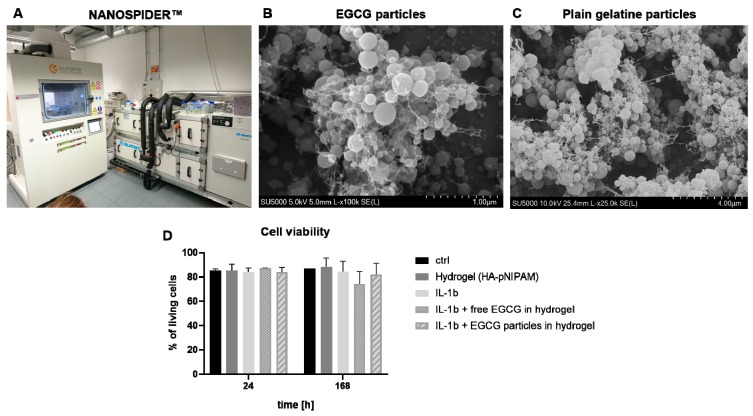
Industrial electrospraying of EGCG particles. (**A**) Electrospinning device NANOSPIDER™; (**B**) representative SEM image of EGCG particles produced using NANOSPIDER™ technology; (**C**) representative SEM image of plain gelatin particles produced by NANOSPIDER™ technology; (**D**) cytocompatibility of the EGCG delivery system (= upscaled EGCG particles in the hydrogel) in 3D alginate beads treated with 5 ng/mL IL-1β for 24 h or seven days (*n* = 1–5, Kruskal–Wallis with Dunn’s post hoc test, * *p* < 0.05, ** *p* < 0.01, *** *p* < 0.001).

Optimizations of needle-free NANOSPIDER™ technology were performed by varying the solution viscosity, electrode distance, spraying solution pH, EMW speed, and temperature, as well as by the addition of a surfactant (Table S1). The final spraying parameters can be found in Table 3. Following these parameters, electrospraying of the mixture of 20% acetic acid, 37.5 µg/mL GA, 5% gelatin, and 0.1% Tween 20 surfactant with or without 2.5 mM EGCG was performed. The resulting particles (Figure 5) had a spherical shape, with small connecting fibers in between, which broke after detaching them from the collector foil. Lower magnification images are shown in Figure S4. The average size of EGCG particles was 2.34 ± 0.3 μm (*n* = 3 final batches), while particles without EGCG showed an average size of 3.83 μm (one batch). The resulting EGCG microparticles were then dispersed in HA-pNIPAM and the cytocompatibility of this formulation was evaluated in the 3D disc disease model (Figure 5D). Cells in alginate beads (*n* = 1–5 donors) were stimulated with 5 ng/mL IL-1β alone or in the presence of the EGCG delivery system (IL-1β + EGCG particles in the hydrogel) or free EGCG in the hydrogel. Hydrogel alone and untreated beads were used as controls. Cell viability after 24 h and seven days was not significantly altered in either experimental group.

## 4. Discussion

During DDD, regulation of the IL-1 pathway is altered, activating inflammatory cascades. At the same time, the expression of ECM-degrading enzymes significantly increases [33]. Recently, EGCG has been highlighted as a promising therapeutic compound due to its anti-inflammatory and anticatabolic activities [4,6]. Therefore, this study explored a novel EGCG microencapsulation method for intradiscal delivery in the treatment of DDD. The production of EGCG microparticles by electrospraying was successfully optimized, the particles were characterized in terms of cytocompatibility and EGCG activity, and the particle production was scaled up for potential industrial use/clinical application. The study also provided a proof of concept that EGCG activity/release in the disc could be favorably modulated when combining EGCG microparticles with a suitable carrier. In order to allow for future clinical implementation, the envisioned intradiscal drug delivery system should possess the following characteristics: (1) biocompatibility, (2) tissue retention, (3) therapeutic activity, (4) biodegradability, (5) minimally invasive applicability, and (6) sustained release.

The cytocompatibility of both electrosprayed GA–gelatin and EGCG–GA–gelatin microparticles was confirmed. In previous studies, which tested gelatin-based materials in disc regeneration, the injection of pure gelatin microspheres (without cells or drugs) was shown to positively affect the NP microenvironment and decrease the rate of apoptosis in a rabbit disc degeneration model [34,35]. Gelatin microspheres were also used to deliver platelet-rich plasma (PRP) into the NP in a rabbit model, providing sustained PRP release through diffusion via the pores of the slowly degrading gelatin network [36]. It was also demonstrated that the microencapsulation of EGCG into gelatin enhances EGCG stability by delaying its dissolution in aqueous media and by the formation of the interactions between the active molecule and its encapsulating matrix [17]. Therefore, gelatin can be considered suitable for the encapsulation of EGCG.

To ensure tissue retention and reduce particle displacement and damage, microparticles were dispersed in an in situ-forming biomaterial, HA-pNIPAM, with a gelation temperature of around 30 °C, thereby quickly solidifying upon injection into the disc [21]. HA-pNIPAM has been successfully tested for minimally invasive intradiscal delivery of cells and growth factors in preclinical settings [37,38,39,40] and injected in vivo [41]. In the IVD, HA-pNIPAM can be broken down in vivo via the degradation of HA. Although high Mw pNIPAM is not biodegradable [42], short pNIPAM chains (Mw < 100,000) were shown to undergo renal excretion in vivo [43]. The potential incorporation of linkers might enable the generation of small Mw degradation products for improved renal excretion, hence increasing the clinical applicability of HA-pNIPAM. 

The active dose of EGCG in various cell types was reported between 5 and 500 µM [13,44]. Given the fact that diurnal fluid loss in the human NP is 10–20% [45,46] and burst release may occur [14], high-dose (mM) EGCG was chosen in our study, aiming for a stable release of 10–100 µM EGCG over long periods of time (e.g., one month). The biological activity of EGCG after electrospraying was analyzed by gene and protein expression of EGCG targets in IVD cells [4]. After one week in the pro-inflammatory 3D cell cultures, EGCG microparticles strongly inhibited IL-1β-dependent expression of IL-6, IL-8, COX-2, MMP1, MMP3, and MMP13, providing evidence that EGCG activity was retained after electrospraying. Although a proof of concept was provided, the release pattern (e.g. detailed evaluation of burst release) and the biological activity of EGCG microparticles should be further tested and verified on a larger scale. 

It has been reported that up to 3 mL of material can be injected into the center of human lumbar degenerated NP [47,48]. Therefore, EGCG microparticles were mixed with 1 mL HA-pNIPAM. Our data suggested that such a drug delivery system could provide sustained release. As the relationships between the concentration of encapsulated EGCG, the quantity of polymers, and the EGCG release rates in vivo are not yet known, the amounts of these components may need to be readjusted upon testing in organ culture models, to achieve optimal EGCG activity and good biomechanical properties. The drug release rate can be controlled, e.g. by gelatin crosslinking or using specific particle sizes.

Hybrid and composite/blend scaffolds have emerged as promising biomaterials, e.g. for cartilage tissue engineering [49]. Nano/microcomposite structures allow for modulating the material’s degradation rate, mechanical properties, and drug release rate. We envision that EGCG microparticles embedded in the slowly biodegradable material will favor IVD health by downregulating local inflammation, oxidative stress, and catabolism. As therapeutic interventions using anti-inflammatory and anti-catabolic compounds are thought to restore the homeostasis and functionality of the resident disc cells, this strategy could be useful in the treatment of moderately painful DDD. In moderate DDD, homeostasis in the NP has shifted towards catabolism and cytokines are released from disc cells, irritating adjacent nerves. At the same time, the proteoglycan content in the NP remains sufficient [50]. To ensure successful clinical translation, the safety and efficacy of the formulation should be tested in an animal model that accurately mimics these conditions. Therefore, in the future, the proposed therapeutic strategy will be tested in dogs with experimentally induced and/or naturally occurring mild/moderate IVD degeneration [51,52]. On the other hand, the proposed therapeutic strategy may not be suitable as a single treatment for the advanced stages of DDD, where proteoglycans of the NP are degraded and disc cells are disappearing. Nevertheless, once optimized and fully characterized, our proposed microparticle-based delivery system will likely be compatible with other small-molecular therapeutics, hence offering great clinical versatility/applicability.

## 5. Conclusions

EGCG is a promising compound for the treatment of DDD. However, EGCG is prone to rapid degradation and/or release, which hinders the clinical translation of EGCG-based therapeutics. Several encapsulation techniques have been used to improve EGCG stability and/or prolong its release upon oral administration, but an encapsulation of EGCG for the treatment of DDD remained thus far unexplored. This study successfully optimized the electrospraying-based production of active and biocompatible EGCG microparticles and provided a proof of concept that combining the developed EGCG microparticles with a suitable carrier could favorably modulate EGCG activity/release in the disc. In addition, the industrial-scale microencapsulation of EGCG for further (pre)clinical development was optimized. Future studies will focus on thoroughly investigating the biological activity of the EGCG microparticle-based drug delivery system in human disc cells and organ culture models, to determine the optimal mode and duration of its therapeutic activity.

## Figures and Tables

**Table 1:** Target genes and primer Assay ID numbers used in this study.

Target Gene	Assay ID	Function
GAPDH	Hs02758991_g1	housekeeping gene
IL-6	Hs00174131_m1	inflammation mediator
IL-8	Hs00174103_m1	inflammation mediator
COX-2	Hs00153133_m1	pain mediator
MMP1	Hs00233958_m1	cleaves mainly collagens (I, II, III)
MMP3	Hs00968305_m1	cleaves proteoglycans and collagens (II, III)
MMP13	Hs00233992_m1	cleaves mainly collagens (I, II, III)

**Table 2:** The release of IL-6 and IL-8 in IVD cells cultured in 3D for seven days in the presence of 5 ng/mL IL-1β with and without EGCG microparticles (*n* = 3 repeats labelled as particles (1), (2) and (3), *t*-test IL-1β vs. IL-1β + EGCG particles, * *p* < 0.05, ** *p* < 0.01, *** *p* < 0.001). bdl = below detection limit. ctrl = untreated cells.

Target	ctrl	IL-1β	IL-1β+EGCG Particles (1)	IL-1β+EGCG Particles (2)	IL-1β+EGCG Particles (3)
IL-6	12 ± 20 pg/mL	376 ± 125 ng/mL	bdl	179 ± 311 pg/mL	224 ± 389 pg/mL
IL-8	bdl	269 ± 188 ng/mL	bdl	bdl	bdl

**Table 3:** Final parameters chosen for electrospraying using NANOSPIDER™ technology.

**Cylinder diameter**	0.5 mm
**Wire speed**	70 mm/min
**EMW speed**	400 mm/s
**Electrode distance**	250 mm
**Positive voltage**	60 kV
**Negative voltage**	–10 kV
**Relative humidity**	40%
**Temperature**	12 °C

## Supplementary Materials

The following are available online at https://www.mdpi.com/1999-4923/11/9/435/s1, Figure S1: Scanning electron microscopy images of electrosprayed 4% and 6% gelatin particles crosslinked with 37.5 µg/mL, 62.5 µg/mL, and 87.5 µg/mL glutaraldehyde (GA) and sprayed at flow rates 2 µL/min and 4 µL/min. Figure S2: The release of IL-6 from disc cells cultured in 3D for seven days in the presence of 5 ng/mL IL-1β with and without EGCG microparticles. Figure S3: Korsmeyer–Peppas model of drug release kinetics. Figure S4: Representative SEM image of EGCG particles produced using NANOSPIDER™ technology in 10k magnification and 5k magnification. Table S1: Examined electrospraying parameters (NANOSPIDER™ technology).
