# Supplementary material for: Electrospray-Based Microencapsulation of Epigallocatechin 3-Gallate for Local Delivery into the Intervertebral Disc
Source: Pharmaceutics. 2019 Sep 1;11(9):435. doi: 10.3390/pharmaceutics11090435 (PMC6781552; doi:10.3390/pharmaceutics11090435)
Supplement: Supplementary file 1 [file pharmaceutics-11-00435-s001.pdf]

pharmaceutics

MDPI

# Supplementary Materials: Electrospray-Based Microencapsulation of Epigallocatechin 3-Gallate for Local Delivery into the Intervertebral Disc

Moira Loepfe, Anja Duss, Katerina-Alexandra Zafeiropoulou, Oddny Björgvinsdóttir, Matteo D'Este, David Eglin, Giuseppino Fortunato, Juergen Klasen, Stephen J. Ferguson, Karin Wuertz-Kozak and Olga Krupkova

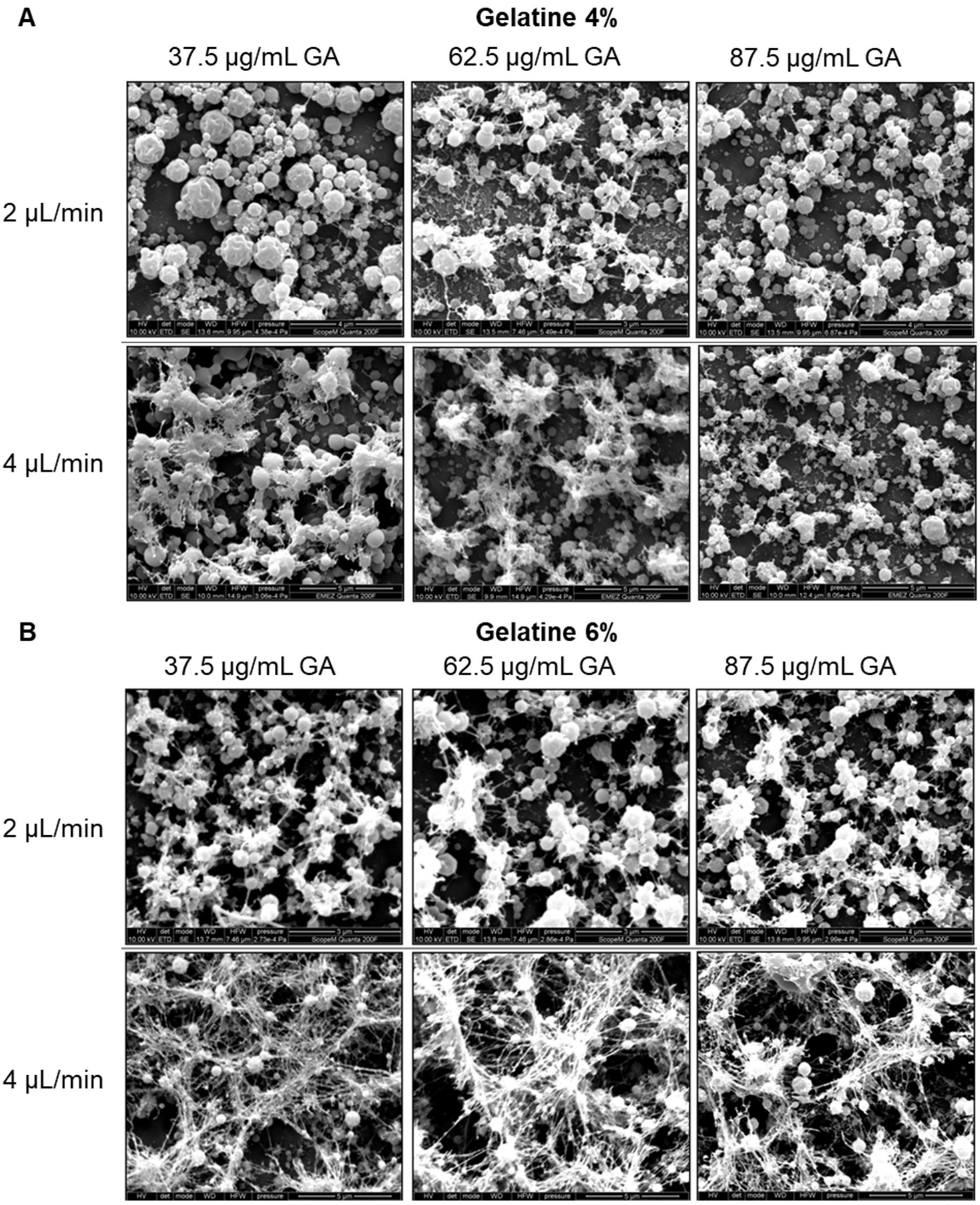


**Figure S1.** Scanning electron microscopy images of electrosprayed (**A**) 4 % and (**B**) 6 % gelatine particles crosslinked with 37.5 µg/mL, 62.5 µg/mL, and 87.5 µg/mL Glutaraldehyde (GA) and sprayed at flow rates 2 µL/min and 4 µL/min.

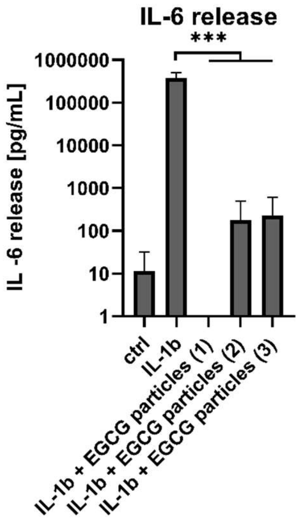


**Figure S2.** The release of IL-6 from disc cells cultured in 3D for seven days in the presence of 5 ng/mL IL-1β with and without EGCG microparticles (n = 3 repeats, t-test IL-1β vs. IL-1β + EGCG particles, *p < 0.05, ** p < 0.01, ***p < 0.001).

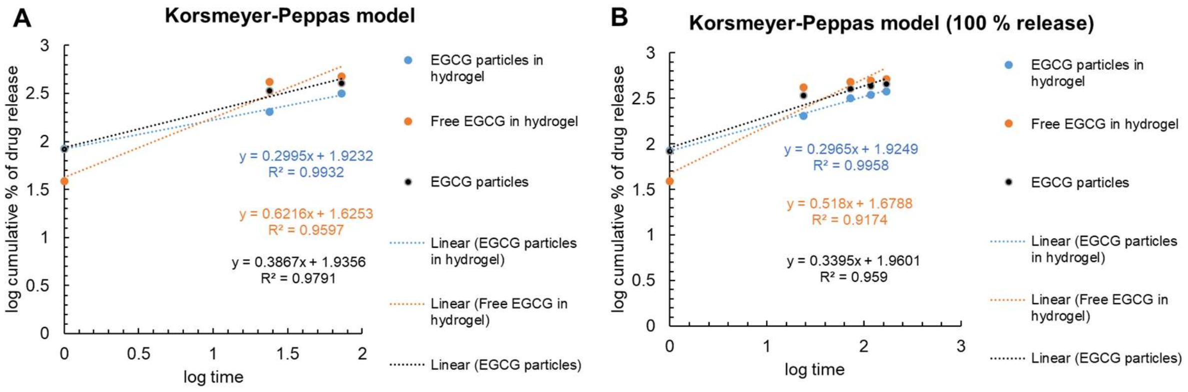


**Figure S3.** Korsmeyer-Peppas model of drug release kinetics. **(A)** First ~60–80% of EGCG release in each group was plotted as log cumulative % drug release vs. log time. Diffusion coefficient (n) was determined from the respective equations. **(B)** Korsmeyer-Peppas plot for 100% release was shown for comparison. Orange = free EGCG in hydrogel, blue = EGCG particles in hydrogel, black = EGCG particles.

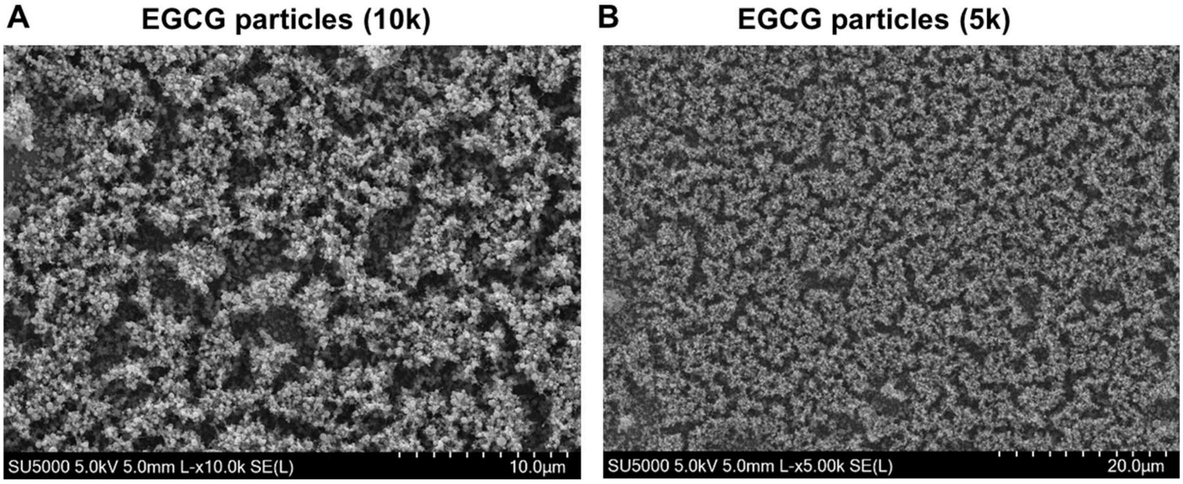


**Figure S4.** Representative SEM image of EGCG particles produced using NANOSPIDER™ technology in **(A)** 10k magnification and **(B)** 5k magnification.

**Table S1.** Examined electrospraying parameters (NANOSPIDER™ technology). Heated = the gelatine mix has been heated inside an Erlenmeyer flask attached to a reflux condenser by lowering the flask into an oil bath which was warmed by a heating plate to 110°C with 500 rpm. Cooled = the gelatine mix has been cooled to 4°C in the fridge.

| Trial | Gelatine content (%) | Cylinder diameter (mm) | EMW speed (mm/s) | Electrode distance (mm) | Humidity (%) | Temperature (°C) |
|---|---|---|---|---|---|---|
| 1 | 4 | 0.7 | 290 | 250 | 40 | 20 |
| 2 | 4 | 0.5 | 260 | 200 | 40 | 20 |
| 3 | 4 | 0.5 | 600 | 200 | 40 | 20 |
| 4 | 4 | 0.7 | 200 | 200 | 40 | 15 |
| 5 | 4 | 0.7 | 200 | 200 | 40 | 5 |
| 6 | 10 | 0.5 | 200 | 250 | 40 | 11 |
| 7 | 6 | 0.5 | 280 | 250 | 40 | 11 |
| 8 | 6 | 0.5 | 280 | 250 | 10 | 10 |
| 9 | 6 | 0.5 | 280 | 250 | 60 | 10 |
| 10 | 6 | 0.5 | 400 | 250 | 60 | 10 |
| 11 | 6 | 0.5 | 400 | 250 | 60 | 20 |
| 12 | 5 (heated 24h) | 0.5 | 400 | 250 | 40 | 12 |
| 13 | 5 (heated 48h) | 0.5 | 400 | 250 | 40 | 12 |
| 14 | 4.5 | 0.5 | 400 | 250 | 40 | 12 |
| 15 | 5 (cooled at 4°C) | 0.5 | 400 | 250 | 40 | 12 |
| 16 | 10 (heated 48h) | 0.5 | 400 | 250 | 40 | 12 |
| 17 | 7.5 (heated 48h) | 0.5 | 400 | 250 | 40 | 12 |
| 18 | 5 (no NaOH, Tween20) | 0.5 | 400 | 250 | 40 | 12 |
| 19 | 5 (Tween20) | 0.5 | 400 | 250 | 40 | 12 |
| 20 | 10 (heated 36h, Tween20) | 0.5 | 400 | 250 | 40 | 12 |
